# 用于*N*-连接完整糖肽鉴定的糖基化蛋白质组学数据处理方法最新研究亮点

**DOI:** 10.3724/SP.J.1123.2022.04022

**Published:** 2022-06-08

**Authors:** Zhen LIU

**Affiliations:** 南京大学化学化工学院, 生命分析化学国家重点实验室, 江苏 南京 210023

蛋白质糖基化与疾病的发生发展密切相关,临床上使用的大多数肿瘤标志物是糖基化蛋白质。在组学层次上进行位点特异性糖型的分析对发现新型疾病标志物、提高基于蛋白质糖基化的精准医学研究水平等具有重要作用。色谱-质谱联用技术在糖蛋白的分离分析研究中得到了广泛的应用。基于液相色谱-串联质谱(liquid chromatography-tandem mass spectrometry, LC-MS/MS)的完整糖肽鉴定已成为研究蛋白质上位点特异性糖链修饰的主要手段,其主要优势在于分析过程中可以同时揭示蛋白位点与糖链修饰的信息,从而在组学层次实现规模化的蛋白糖基化分析。但是,与其他翻译后修饰相比,糖基化具有组成复杂、相对分子质量分布范围广的特点,因此完整糖肽的数据库检索空间较大。同时完整糖肽的质谱谱图较为复杂。在碰撞诱导碎裂的完整糖肽质谱谱图中,糖链产生的碎片离子强度较高,而肽段骨架碎裂不完全,产生的碎片离子强度较低,给完整糖肽的谱图解析带来了困难。因此,发展具有高灵敏度与可信度的质谱谱图鉴定方法是深度解析蛋白糖基化修饰的关键环节。

针对上述问题,近日,中国科学院大连化学物理研究所的叶明亮课题组开发了一款非糖库依赖的*N*-糖肽谱图鉴定软件Glyco-Decipher用于位点特异性糖型的深度解析^[1]^。该方法首先匹配*N*-连接糖链的五糖核心结构,确定肽段的相对分子质量,通过理论去糖峰实现肽段骨架的初步鉴定。之后利用肽段骨架的碎裂规律,基于碎裂特征的模式识别,发展了谱图拓展策略进一步提高完整糖肽的鉴定灵敏度。该方法不仅显著提高了完整糖肽谱图的解析效率,而且实现了肽段骨架碎裂不完全的完整糖肽的鉴定,在理论去糖峰方法的基础上将完整糖肽的鉴定数目提升了31%,提高了揭示糖基化修饰微观不均一性的能力。与糖肽谱图解析的其他方法相比,完整糖肽的谱图解析率提升了34%~179%。该方法在谱图解析过程中不依赖于已有的糖链数据库信息,大大减小了完整糖肽的检索空间。同时可以全面系统地揭示样品中糖链修饰的相对分子质量分布,发现未收录在数据库的新型糖链。发展的单糖步进策略利用连续碎裂的糖链离子,确定未知糖链的组成及其上所带有的化学修饰或生物修饰。在鉴定可信度方面,综合考虑糖链的相对分子质量以及碎片离子信息,在谱图层次将完整糖肽的鉴定假阳性率控制在1%以内。此外,基于一级谱的流出曲线信息,实现糖肽的无标记定量,可在样品层次或具体蛋白位点层次方面考察糖链修饰的丰度分布,为后续的生物学研究提供了有力工具。

该方法的优越性主要体现在5个方面:(1)通过*N*-糖链的五糖核心特征结构确定肽段骨架的相对分子质量,采取非糖库依赖的肽段优先鉴定策略,大大减小了完整糖肽的检索空间;(2)利用肽段骨架的碎裂特征规律,发展了谱图拓展策略,实现了低丰度或肽段碎裂不完全糖肽的鉴定;(3)非糖库依赖谱图解析实现了数据库之外未知糖链(如带修饰糖链)的发现与鉴定;(4)鉴定过程中该方法能够系统全面地考虑肽段与糖链的碎片离子,保证了完整糖肽的鉴定可信度;(5)位点特异性糖型的深度解析提高了糖基化修饰的位点占有率等定量分析考察的准确度。

上述工作通过利用肽段骨架碎裂规律的非糖库依赖检索策略,在*N*-糖肽质谱数据处理方面获得了目前最高的谱图解析效率,实现了深度准确的位点特异性糖型分析,为糖生物学功能研究提供了新工具。
